# Evolution of the income-related gap in health with old age: evidence from 20 countries in European and Chinese panel datasets

**DOI:** 10.1007/s10433-023-00781-y

**Published:** 2023-08-10

**Authors:** Mengling Cheng, Nicolas Sommet, Daniela S. Jopp, Dario Spini

**Affiliations:** 1 Swiss Centre of Expertise in Life Course Research, Faculty of Social and Political Sciences, University of Lausanne, Eopolis 5797, CH-1015 Lausanne, Switzerland; 2 Institute of Psychology, University of Lausanne, Lausanne, Switzerland

**Keywords:** Income-related health gap, Age-as-leveller, Cumulative advantage/disadvantage, Old age, Cross-national study, Longitudinal analysis, Causal inference

## Abstract

Some studies show that the protective effect of higher income on health *weakens* with old age (age-as-leveller pattern), whereas others show that it *strengthens* with old age (cumulative advantage/disadvantage pattern). Many existing studies are limited in that they use single-country and/or single-timepoint designs. To overcome these limitations and better understand how the income-health gradient evolves in older age, we used cross-national and longitudinal data of the Survey of Health, Ageing and Retirement in Europe (2004–2019, *N* = 73,407) and the China Health and Retirement Longitudinal Study (2011–2018, *N* = 10,067). We operationalised health using multimorbidity and three alternative indicators (functional disability, mobility disability, and memory). We performed Poisson growth curve modelling to capture the between-participant effects of age and the within-participant effects of aging. We obtained three consistent and robust findings for Europe (patterns were observed in most countries) and China. First, the protective effect of higher income on multimorbidity, functional disability, and mobility disability was weaker for older than for younger adults (between-participant age-as-leveller pattern). Second, only the protective effect of higher income on mobility disability weakened over the later life course (within-participant age-as-leveller pattern). Third, the protective effect of higher income on memory was stronger for older than for younger adults and strengthened over the later life course (between-participant and within-participant cumulative advantage/disadvantage pattern). Longitudinal data, growth curve modelling distinguishing the between-participant from within-participant effect, and adjustments for potential confounders based on the hypothesised causal structure enabled us to better navigate the landscape of causal inference. Findings suggest that the income-related gap in physical health but not in cognitive health narrows in old age for both Europe and China.

**Supplementary Information:**

The online version contains supplementary material available at 10.1007/s10433-023-00781-y.

## Introduction

Existing studies testing how the link between income and health evolves with old age document two opposite patterns of findings (Holman and Walker 2021): the age-as-leveller pattern and the cumulative advantage/disadvantage pattern. Studies in line with the age-as-leveller pattern report that the protective effect^1^ of higher income on health *weakens* with old age (Bonaccio et al. 2019; Brown et al. 2016; Crimmins et al. 2004; Griffith et al. 2021; House et al. 1990; Kim and Durden 2007; Schöllgen et al. 2010; Sieber et al. 2020). Scholars believe that the fundamental reason for this trend is that the socioeconomic determinants of health are outweighed by biological determinants of health after a certain point in later life (Hoffmann 2011; Rehnberg 2020) and the selective mortality (Dupre 2007), meaning that income becomes less and less predictive of health as people age. In contrast, studies in line with the cumulative advantage/disadvantage pattern report that the protective effect of higher income on health *strengthens* with old age (Chen et al. 2010; Crystal and Shea 1990; Dannefer 2018; Lahelma et al. 2015; Leopold 2018; O’Rand 2002; Veenstra and Aartsen 2022; Willson et al. 2007). Scholars believe that the fundamental reason for this trend is that socioeconomic-related advantage/disadvantage accumulate over the life course (DiPrete and Eirich 2006; Lynch 2003), meaning that income becomes increasingly predictive of health as people age.

Many existing studies are limited in that (i) they use single-country designs and (ii) they use single-timepoint designs. First, most of the existing studies were conducted in *a single country*, most of the time in the U.S. (for example, House et al. 1990; Kaplan et al. 2007; for an exception, see Sieber et al. 2020). Generalization of findings from such studies may be misleading (Rai et al. 2013) because health-related estimates are known to vary from one national setting to another and should always be interpreted in a context-sensitive manner (Kessler and Bromet 2013). Studies using cross-national designs and replications in different countries are therefore needed to better understand how the link between income and health evolves with old age. Second, most of the existing studies used *single-timepoint designs*, that is, they focused on a particular year of data collection (for example, Lowry and Xie 2009; Robert et al. 2009; for an exception, see Rehnberg et al. 2019). Findings from such studies may also be misleading (Galbraith et al. 2017) because their health-related estimates pertain to comparisons between participants of different ages and cannot be interpreted as corresponding to changes in health over time (Fitzmaurice et al. 2011). Studies using longitudinal designs to capture within-participant dynamics over time are therefore needed to better understand how the link between income and health evolves with old age.

In the present study, we investigated in two older adult panel datasets how the effect of income on health evolves with age from both a cross-national and a longitudinal perspective. In contrast to most existing studies that focused on the U.S., we chose to focus on two important economies where—in recent decades—population aging has put a strain on health systems and social services, namely, Europe and China (Rechel et al. 2013; Zhao et al. 2014). Moreover, in contrast to most existing studies that used cross-sectional data, we used longitudinal European and Chinese datasets on older adults, namely, the Survey of Health, Ageing and Retirement in Europe (SHARE) and the China Health and Retirement Longitudinal Study (CHARLS). Our study’s aim is to make causal inferences about how the effects of income on health evolve with age. We tested the following two hypotheses: (i) the effect of income on health weakens as people age (the age-as-leveller hypothesis) or (ii) the effect of income on health strengthens as people age (the cumulative advantage/disadvantage hypothesis). We operationalised health using an indicator particularly relevant to old age, namely, multimorbidity (Makovski et al. 2019). To gain further evidence, we also used three alternative measures of outcomes from physical and cognitive health domains, namely, functional disability, mobility disability, and memory. 

## Methods

### Study design

Our study aimed to make causal inferences, examining how the effects of income on health evolve with age. Our approach to causality involved using longitudinal data (VanderWeele et al. 2016), growth curve modelling distinguishing the between-participant effect from within-participant effect (Raymaekers et al. 2020), and adjustments for potential confounders based on the “hypothesised causal structure” (Wysocki et al. 2022).

### Samples

We chose to work with longitudinal data because longitudinal data facilitates causal inference (VanderWeele et al. 2016). We used cross-national data from two different sources: SHARE and CHARLS. Both SHARE and CHARLS are longitudinal surveys that offer (i) prospective data (e.g., recurring health assessment, income), and (ii) retrospective data (e.g., childhood sociodemographic information). Specifically, SHARE is composed of a series of nationally representative panel surveys conducted biennially since 2004 that collected health data on approximately 140,000 people aged 50 or older from 28 European countries and Israel. CHARLS is a nationally representative panel survey conducted biennially since 2011 that collected the same kind of health data on approximately 17,500 Chinese residents aged 45 or older.

We used all available waves of the SHARE data (i.e., six waves spanning 2004 to 2019) and the CHARLS data (i.e., four waves spanning 2011–2018). In SHARE, older adults are defined as people aged 50 or older, whereas in CHARLS they are defined as aged 45 or older. In both cases, we removed the few observations that were incorrectly included in the sample (i.e., in SHARE, people < 50 years old; in CHARLS, people < 45 years old). We included eligible observations based on two criteria: (i) complete cases for health and sociodemographic variables (a total of 277,633 observations in SHARE; a total of 37,451 observations in CHARLS) and (ii) at least two waves to be able to estimate within-participant effects over time (87.60% of observations in SHARE; 97.43% in CHARLS). Our final sample in SHARE comprised 243,207 observations from 73,407 older adults in 19 countries, and our final sample in CHARLS comprised 36,487 observations from 10,067 older adults (for the characteristics of both samples, see Table 1).

**Table 1:** Description of the SHARE (2004–2019) and CHARLS (2011–2018) samples

	SHARE (2004–2019)	CHARLS (2011–2018)
Men (%)	44.31	48.25
Mean age (in years)	66.9 (*SD* = 9.5)	61.3 (*SD* = 9.4)
Urban residence (%)	69.25	33.57
Currently married (%)	73.14	87.35
Currently not working (%)	64.65	33.50
Mean household size (in persons)	2.3 (*SD* = 1.0)	3.5 (*SD* = 1.7)
Mean equivalized income (in 2010 USD)	38,405 (*SD* = 65,133)	2,094 (*SD* = 7,241)
Mean household wealth (in 2010 USD)	35,947 (*SD* = 140,283)	312 (*SD* = 65,022)
Educational level (%)		
Less than upper secondary education	42.48	90.86
Upper secondary or vocational education	36.61	8.12
Tertiary education	20.91	1.02
Multimorbidity (%)	45.43	43.78
Mean number of chronic diseases	1.9 (*SD* = 1.5)	1.8 (*SD* = 1.5)
Mean number of functional disabilities	0.1 (*SD* = 0.3)	0.5 (*SD* = 0.4)
Mean number of mobility disabilities	1.3 (*SD* = 1.5)	1.5 (*SD* = 1.4)
Mean number of immediate word recall	5.2 (*SD* = 1.5)	3.8 (*SD* = 1.3)

Harmonized educational levels provided by the SHARE and CHARLS data are used in the study

### Measures

#### Equivalized income decile (time-constant)

Participants reported their total household income during the last year in both SHARE and CHARLS. Potential sources of income were earnings, capital, pension, government transfers, and other sources. To account for inflation, we converted the total household income into inflation-adjusted total household income. Specifically, we used the annual consumer price index available for each year of the survey as an inflation multiplier (i.e., we divided household income by the year-specific consumer price index included in the datasets). To adjust for the difference in household size, we converted inflation-adjusted total household income into equivalized income. Specifically, we used the square root equivalence scale of the Organization for Economic Co-operation and Development (OECD 2013), namely, we divided the inflation-adjusted total household income by the square root of household size (for descriptive statistics, see Table 1). As the income of older adults changes marginally over time in our datasets (for SHARE, 65%-68% of participants remain in the same income decile wave-to-wave; for CHARLS, 63%-68% of participants remain in the same income decile wave-to-wave),^2^ we treated equivalized income as a time-constant variable. To compare estimates across national contexts that use different currencies and/or have varying levels of economic development, we created a variable of equivalized income decile for each participant (1 = *bottom 10%*; 10 = *top 10%*).

#### Multimorbidity (time-varying)

We operationalised later-life health by counting the number of chronic diseases present within the individuals in each wave (Marengoni et al. 2011). Participants reported whether they had been diagnosed by a doctor with any of 12 chronic diseases in SHARE (i.e., high blood pressure, diabetes, cancer, lung disease, heart disease, stroke, arthritis, high cholesterol, ulcer, Parkinson disease, cataracts, or hip fracture) and 12 chronic diseases in CHARLS (i.e., high blood pressure, diabetes, cancer, lung disease, heart disease, stroke, arthritis, dyslipidemia, liver disease, kidney disease, stomach/digestive disease, or asthma).

#### Covariates

In our models, we controlled for variables based on the “hypothesised causal structure” (Wysocki et al. 2022). Specifically, we proposed a causal structure in which socioeconomic and demographic factors are plausible confounders of the causal effect of income on later-life health. The following variables have been evidenced to be confounders of income and later-life health: wealth, education, working status, gender, residence region, marital status, and household size (Brown et al. 2016; Chen et al. 2010; Hoffmann 2011; Lahelma et al. 2015; Leopold 2018; Sieber et al. 2020; Veenstra and Aartsen 2022). We included these control variables in our models: wealth decile (from 1 = *bottom 10%* to 10 = *top 10%*), education level (1 = *less than upper secondary*, 2 = *upper secondary or vocational*, 3 = *tertiary*), gender (-0.5 = *men*, + 0.5 = *women*), region of residence (0 = *urban*, 1 = *rural*), current marital status (0 = *not married*, 1 = *married*), current working status (0 = *not working*, 1 = *working*), and household size (i.e., the number of people living in the household).

#### Alternative measures of health outcomes for supplementary analyses

In our supplementary analyses, we used three alternative measures of health outcomes from two health domains: for the physical health domain, we used functional disability and mobility disability; for the cognitive health domain, we used memory. Specifically, (i) we measured functional disability using the number of instrumental activities of daily living reported to be difficult out of the following: using the phone, managing money, and taking medications; (ii) we measured mobility disability using the number of activities reported to be difficult out of the following: walking 100 m, climbing several flights of stairs, getting up from a chair, stooping or kneeling or crouching, extending arms up, lifting 10 pounds (in SHARE) or 5 kg (in CHARLS), and picking up a small coin; and (iii) we measured memory using the number of words from a 10-word list that were correctly recalled immediately.

### Analytic strategy

#### Poisson growth curve models

To take the hierarchical structure of the data into account and estimate health trajectories over the later life course, we built a series of multilevel growth curve models. Regarding SHARE, we treated wave-specific observations (*N* = 243,207 level-1 units) as nested in participants (*K* = 73,407 level-2 units) and countries (*L* = 19 level-3 units). Regarding CHARLS, we treated wave-specific observations (*N* = 36,487 level-1 units) as nested within participants (*K* = 10,067 level-2 units). We used Poisson regression rather than negative binomial regression because the overdispersion test did not reject the null hypothesis of equidispersion, *χ*^*2*^ (3, *N* = 243,207) = 88,814, *p* = 1.00 in SHARE, *χ*^*2*^ (2, *N* = 36,487) = 14,135, *p* = 1.00 in CHARLS. We built Poisson growth curve modelsrather than linear growth curve models because the outcome variables (i.e., multimorbidity, functional disability, mobility disability, and memory) are count variables that follow a Poisson distribution (King 1988).

#### Centering strategy to disentangle the between-participant from the within-participant effect

Between-participant estimates and within-participant estimates may lead to different results (Curran and Bauer 2011) and have different implications in terms of directionality (Allison 2009). To better assess causality, we used an analytical approach that enabled us to estimate both the between-participant effect and the within-participant effect (Raymaekers et al. 2020). Specifically, we used Fairbrother’s (2014) centering strategy and computed two age variables: (i) grand-mean centered mean age and (ii) person-mean centered age. To compute the *grand-mean centered mean age*, we centered each participant’s mean age across all waves on the grand mean age of all participants. This variable enabled us to capture the between-participant effect of age (i.e., the effect of age-related differences between distinct participants). To compute the *person-mean centered age*, we centered each participant’s age in each wave of the survey on their individual mean age across all waves. This variable enabled us to capture the within-participant effect of aging (i.e., the effect of age-related changes within a single participant over time).

#### Focal model equation

We regressed health outcome variables on five focal predictors: (i) grand-mean centered mean age (Age_gmc_i_), (ii) income decile (Income Decile_i(k)_), (iii) grand-mean centered mean age × income decile (to estimate whether the effect of income against multimorbidity differed between younger and older participants), (iv) person-mean centered age (Age_cmc_ij_), and (v) person-mean centered age × income decile (to estimate whether the effect of income against multimorbidity changed as participants aged). We also included a set of seven control variables (see Eq. 1).

1 $$\begin{aligned} {\text{log}}(\lambda_{{{\text{ij}}[{\text{k}}]}} ) \, & = \beta_{00[0]} + \beta_{{0{1}[0]}} \times {\text{ Age}}\_{\text{gmc}}_{{\text{j}}} + \beta_{{0{2}[0]}} \times {\text{ Income Decile}}_{{{\text{j}}[{\text{k}}]}} \\ & \quad + \beta_{{0{3}[0]}} \times {\text{ Age}}\_{\text{gmc}}_{{{\text{j}}[{\text{k}}]}} \times {\text{ Income Decile}}_{{{\text{j}}[{\text{k}}]}} + \, \left( {\beta_{{{1}0[0]}} + u_{{{\text{1i}}[{\text{k}}]}} } \right) \, \\ & \quad \times {\text{ Age}}\_{\text{cmc}}_{{{\text{ij}}[{\text{k}}]}} + \beta_{{{11}[0]}} \times {\text{ Age}}\_{\text{cmc}}_{{{\text{ij}}[{\text{k}}]}} \times {\text{ Income Decile}}_{{{\text{j}}[{\text{k}}]}} \\ & \quad + \beta_{{{\text{ij}}[{\text{k}}]}} \times {\text{ Control}}_{{{\text{ij}}[{\text{k}}]}} + u_{{0{\text{j}}\left[ {\text{k}} \right]}} [ + u_{{00{\text{k}}}} ] \\ \end{aligned}$$

where *Y*_ij[k]_ is the outcome, which follows a Poisson distribution (*Y*_ij[k]_ ~ Poisson(*λͅ*_ij[k]_)); *i* = 1, 2, …, *N* (wave-specific observations); *j* = 1, 2, …, *K* (participants); *β*_ij[k]_ × Control_ij[k]_ represents the vector of the seven control variables (i.e., see the relevant subsection in “Measures”);* u*_0j[k]_ represents the participant-level residuals; and *u*_1i[k]_ represents the random slope of age. In SHARE, the equation involves three levels, including the terms and subscripts in brackets, where *k* = 1, 2, …, *L* (countries), and *u*_00k_ represents the country-level residuals. We chose to assess the interaction between age and income decile on the multiplicative scale based on theoretical and methodological reasons. From a theoretical perspective, relative risk measures on the multiplicative scale may be more suitable to assess causality (Poole 2010). From a methodological perspective, relative risk measures on the multiplicative scale have less heterogeneity in statistical significance (VanderWeele and Knol 2014) and offer acceptable effectiveness (Sommet and Morselli 2017).

An interaction term is significant on the multiplicative scale if the combined effect of two exposures is larger or smaller than the product of the individual effects of the two exposures (Knol et al. 2011). In our model, decomposition of the between-participant-based interaction term Age_gmc_j[k]_ × Income Decile_j[k]_ enables us to compare the effect of income decile between younger participants and older participants, whereas decomposition of the within-participant-based interaction term Age_cmc_ij[k]_ × Income Decile_j[k]_ enables us to compare the effect of income decile as participants age over the later life course.

We ran the Poisson growth curve models described above using the glmer function from the lme4 package (version 1.1–26) (Bates et al. 2015) in R (version 4.0.2). The instructions for retrieving the datasets and the R scripts to reproduce our findings are available via the Open Science Framework (OSF): https://osf.io/mb8nc/?view_only=b4d526e930594d66a7428db9fbefc4ba.

## Results

### Main analyses

#### Results from Europe

**Fig. 1:**
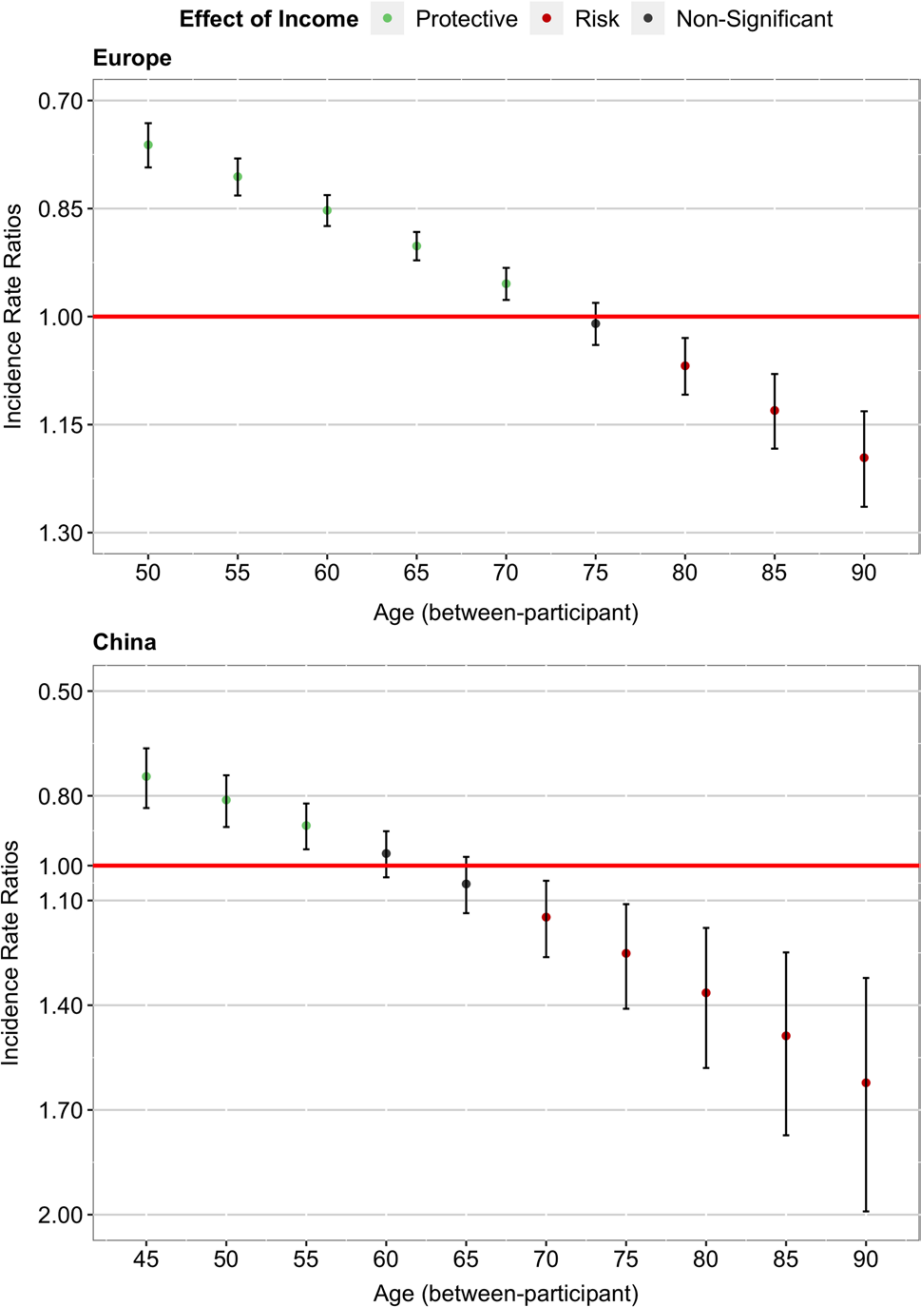
Between-Participant Age-as-Leveller Effect of Income on Multimorbidity in Europe and China. *Note.* Red lines correspond to a null effect; error bars represent 95% CIs.

We observed a significant between-participant age-as-leveller effect (i.e., interaction between grand-mean centered mean age and income decile on the multiplicative scale) for Europe (12 out of 19 countries^3^), IRR = 1.12, 95% CI [1.10, 1.14], *p* < 0.001 (for the full results, see Table 2 left column). Congruent with the age-as-leveller hypothesis, the protective effect of higher income against multimorbidity was weaker for older than for younger adults (for the simple effects of income for each decade of age, see Fig. 1, upper panel). The relationship between income and multimorbidity *reversed* after age 75, meaning that a higher income was no longer protective against multimorbidity but became a risk factor in advanced age.

**Table 2:** Effect of income on multimorbidity as a function of age among older adults in Europe and China

	Europe		China	
	IRRs	95% CI	IRRs	95% CI
Grand-mean centered mean age	1.30***	1.29–1.31	1.12***	1.10–1.14
Person-mean centered age	2.15***	2.03–2.27	2.86***	2.77–2.96
Equivalized income decile (1 = *bottom 10%*, 10 = *top 10%*)	0.91***	0.89–0.93	0.97	0.90–1.03
Grand-mean centered mean age × equivalized income decile	1.12***	1.10–1.14	1.19***	1.12–1.27
Middle-aged adults (-2 SD)	0.74***	0.71–0.78	0.70***	0.62–0.80
Older middle-aged adults (-1 SD)	0.82***	0.80–0.85	0.82***	0.75–0.90
Older adults (+ 1 SD)	1.01	0.98–1.04	1.13**	1.03–1.24
Oldest old adults (+ 2 SD)	1.12***	1.07–1.17	1.33***	1.16–1.52
Person-mean centered age × equivalized income decile	0.99	0.96–1.02	0.99	0.88–1.11
Wealth decile (1 = *bottom 10%*, 10 = *top 10%*)	0.83***	0.81–0.84	0.82***	0.77–0.87
Upper secondary or vocational education	0.91***	0.90–0.93	1.03	0.97–1.10
Tertiary education	0.84***	0.83–0.85	1.07	0.90–1.27
Gender (-0.5 = *men*, + 0.5 = *women*)	1.00	0.99–1.01	1.14***	1.10–1.18
Region of residence (0 = *urban*, 1 = *rural*)	0.99	0.98–1.00	0.95**	0.91–0.99
Current marital status (0 = *not married*, 1 = *married*)	1.00	0.99–1.02	1.04	1.00–1.08
Current working status (0 = *not working*, 1 = *working*)	0.82***	0.81–0.83	0.90***	0.88–0.92
Household size	0.98***	0.98–0.99	0.99**	0.98–0.99
*N*_countries_	19			
*N*_participants_	73,407		10,067	
Observations	243,207		36,487	

IRRs = incidence rate ratios. Comparisons were made between the bottom 10% and the top 10% in terms of income and wealth

*p < 0.05. **p < 0.01. ***p < 0.001

However, we did not observe a significant within-participant age-as-leveller pattern (i.e., interaction between person-mean centered age and income decile on the multiplicative scale) for Europe, IRR = 0.99, 95% CI [0.96, 1.02], *p* = 0.554. In other words, the within-participant protective effect of higher income against multimorbidity *did not* vary over the later life course, meaning that the protection of higher income against multimorbidity was present equally over the later life course of the individual.

#### Results from China

Replicating the results from Europe, we observed a significant between-participant age-as-leveller pattern (i.e., interaction between grand-mean centered mean age and income decile on the multiplicative scale) for China, IRR = 1.19, 95% CI [1.12, 1.27], *p* < 0.001 (for the full results, see Table 2 right column). Again, congruent with the age-as-leveller hypothesis, the protective effect of higher income against multimorbidity was weaker for older than for younger adults (for the simple effects of income for each decade of age, see Fig. 1, lower panel). This time, the relationship between income and multimorbidity *reversed* after age 65, meaning that a higher income was no longer protective against multimorbidity but became a risk factor in old age.

Similar to Europe, we did not observe a significant within-participant age-as-leveller pattern (i.e., interaction between person-mean centered age and income decile on the multiplicative scale) for China, IRR = 0.99, 95% CI [0.88, 1.11], *p* = 0.815. In other words, the within-participant protective effect of higher income against multimorbidity *did not* vary over the later life course, meaning that the protection of higher income against multimorbidity was present equally over the later life course of the individual.

### Supplementary analyses

We conducted three sets of supplementary analyses repeating the main analyses (i) using alternative health outcomes, (ii) using log-transformed equivalized income as our focal predictor, and (iii) assessing the interaction between age and equivalized income decile on the additive scale (i.e., relative excess risk due to interaction [RERI], attributable proportion [AP], synergy index [SI]).

First, we repeated the main analyses using three alternative measures of outcomes from two health domains: functional disability and mobility disability as indicators of the physical health domain and memory as indicator of the cognitive health domain. The supplementary analyses led to consistent findings for both Europe and China (for a summary and comparison with the findings from the main analyses, see Table 3; for the full results for Europe and China, see Tables S1 and S2, respectively). First, between-participant age-as-leveller pattern that was observed in the main analyses was observed in the analyses using functional disability and mobility disability as alternative measures of health outcomes. Second, within-participant age-as-leveller pattern that was *not* observed in the main analyses was observed in the analyses using mobility disability as an alternative measure of health outcome. Third, cumulative advantage/disadvantage pattern that was *not* observed between participants *or* within participants in the main analyses was observed both between participants and within participants in the analyses using memory as an alternative measure of health outcome.

**Table 3:** Patterns of income effects on physical and cognitive health outcomes as a function of age among older adults in Europe and China

	Physical health						Cognitive health	
	Multimorbidity		Functional disability		Mobility disability		Memory	
	IRRs	95% CI	IRRs	95% CI	IRRs	95% CI	IRRs	95% CI
*Europe*								
Between-participant age × income	1.12***	**1.10–1.14**	1.15*	**1.03–1.28**	1.14***	**1.10–1.18**	1.04***	*1.03–1.05*
	**(Age-as-leveller)**		**(Age-as-leveller)**		**(Age-as-leveller)**		*(Cumulative dis/advantage)*	
Within-participant age × income	0.99	0.96–1.02	1.23	0.97–1.55	1.10***	**1.05–1.15**	1.04***	*1.02–1.06*
	(Inconclusive)		(Inconclusive)		**(Age-as-leveller)**		*(Cumulative dis/advantage)*	
*China*								
Between-participant age × income	1.19***	**1.12–1.27**	1.32***	**1.17–1.49**	1.08*	**1.01–1.16**	1.11***	*1.08–1.14*
	**(Age-as-leveller)**		**(Age-as-leveller)**		**(Age-as-leveller)**		*(Cumulative dis/advantage)*	
Within-participant age × income	0.99	0.88–1.11	1.41	0.80–2.47	1.17*	**1.03–1.32**	1.38***	*1.27–1.51*
	(Inconclusive)		(Inconclusive)		**(Age-as-leveller)**		(Cumulative dis/advantage)	

IRRs = incidence rate ratios. Cells in Bold are consistent with the age-as-leveller pattern, cells in Italic are consistent with the cumulative advantage/disadvantage pattern, and cells in underline show inconsistent results. Comparisons were made between the bottom 10% and the top 10% in terms of income

*p < 0.05. **p < 0.01. ***p < 0.001

Second, to ensure a comprehensive analysis, we repeated the main analyses using log-transformed equivalized income instead of income decile as the focal predictor. Similar to the main analyses, the findings in Europe and China exhibited comparable trends: we observed a between-participant age-as-leveller pattern, but did not observe a within-participant age-as-leveller pattern.

Third, in the main analysis, we chose to assess the interaction between age and income decile on the multiplicative scale. In order to gain a comprehensive understanding of the interaction effect between age and income, we also assessed this interaction on the additive scale. An interaction is significant on the additive scale if the combined effect of two exposures is larger or smaller than the sum of the individual effects of the two exposures (Knol et al. 2011). Consistent with the main analyses, the analyses using the additive scale revealed a between-participant age-as-leveller pattern and a within-participant age-as-leveller pattern in Europe and China (for the details of the analysis, see Table S3).

## Discussion

Existing studies testing how the link between income and health evolves with old age are limited in that most of them use single-country and/or single-timepoint designs. In our study, we investigated the evolution of the link between income and health with old age using a cross-national design (involving 20 countries) and a longitudinal design (using two large-scale panel datasets). Our study revealed three main findings that are consistent for Europe and China. These findings were robust to different model specifications.

First, we found between-participant age-as-leveller pattern in multimorbidity, functional disability, and mobility disability. In line with previous cross-sectional studies (e.g., Griffith et al. 2021; House et al. 1990; Robert et al. 2009), the between-participant protective effect of higher income on multimorbidity, functional disability, and mobility disability was weaker for older adults than for younger adults. This could be explained by the selection effect (Pearce and Richiardi 2014), which suggests that lower-income older adults participating in the survey are healthy survivors of mortality selection whose health status is closer to that of higher-income older adults. Therefore, the narrowing health gap observed between higher-income and lower-income older adults may reflect distinct differences between individuals (Ferraro and Farmer 1996) rather than a temporal change in the income–health link over the later life course. In addition, the reversal of the age-as-leveller pattern after age 75 in Europe and after age 65 in China could also be explained by a selection effect. It is plausible that most adults with higher income have a greater likelihood of surviving to an advanced age, while only the most resilient and healthier adults with lower income survive to such age. This is evidenced by the fact that the reversal of the effect was observed in the between-participant analysis (where selection is most potent) and not in the within-participant analysis (where selection is less potent). The earlier onset of the reversal of effect that happened in China, compared with Europe, may be accounted for by the particularly high mortality rates in 1960s to 1980s (Banister and Hill 2004). These high mortality rates were the consequences of the country’s low level of economic and social development during that period (Banister and Zhang 2005).

Second, we only found within-participant age-as-leveller pattern in mobility disability. In line with few longitudinal studies that are available (e.g.Beckett 2000; Sieber et al. 2020), the within-participant protective effect of higher income on mobility disability weakened over the later life course of the individual. One possible explanation for the fact that the between-participant effects were not always replicated when focusing on the within-participant dynamics is that the number of waves in the datasets was somewhat limited (six waves in SHARE and four waves in CHARLS), meaning that statistical power may not have been sufficient to observe small-size longitudinal effects. This further warns us that between-participant results from the literature should be interpreted with caution.

Third, we found both between-participant and within-participant cumulative advantage/disadvantage pattern in memory. In line with previous studies focusing on cognitive health (Cheval et al. 2019; Landy et al. 2017; Lyu and Burr 2016), we found that the protective effect of higher income on memory strengthened over the later life course of the individual. It is possible that the age-as-leveller pattern applies to physical health (i.e., multimorbidity, functional disability, and mobility disability) and that the cumulative advantage/disadvantage pattern applies to cognitive health (i.e., memory). This could be explained by the fact that physical health in later life is more biologically grounded (e.g., changes in aging phenotypes, Fabbri et al. 2015), whereas cognitive health in later life is more socially grounded (e.g., cognitively stimulating activities or experiences, Cullati et al. 2018). As suggested by the lifespan theory, the biological and cultural factors of a health outcome are interwind and the dynamics between biology and culture evolve across the life course (Baltes and Smith 2004), future studies are warranted to investigate the biocultural dynamics underlying a health outcome over the lifespan.

## Limitations and conclusion

One limitation of our study is that our samples were from Europe and China. We chose to work with SHARE and CHARLS because most existing studies were based on U.S. data, and we aimed to study two important economies outside of the U.S. where aging and age-related health issues pose a notable societal challenge. Although our findings were consistent between high-income countries in Europe and a low- and middle-income country, namely, China, replication studies from other countries are needed to determine how the age-as-leveller pattern and the cumulative advantage/disadvantage pattern generalize across different societies (e.g., the Japanese Study of Aging and Retirement [JSTAR] (four waves spanning 2007 to 2013), the Longitudinal Ageing Study in India [LASI] (one wave in 2017/2018), and the Mexican Health and Aging Study [MHAS] (five waves spanning 2001–2018)). Another limitation of our study is that the covariates included in our models were common socioeconomic and demographic variables to both the SHARE and CHARLS data. The inclusion of these variables helped to capture the causal effect of income on later-life health. However, to further mitigate the impact of confounding effects, future studies need to pay more attention to factors that are specific to a certain society, and sensitive to the culture and structure of that society.

Despite these limitations, our use of longitudinal data, growth curve modelling distinguishing the between-participant effect from within-participant effect, and adjustments for potential confounders based on the hypothesised causal structure enabled us to better navigate the landscape of causal inference. We believe that causality should be assessed as a continuum of plausibility as opposed to a dichotomy (for relevant discussion, see Grosz et al. 2020). Our advanced analytical strategy increases the plausibility of causality. Our between-participant and within-participant results suggest that in Europe and China, the income-related gap in physical health—but not cognitive health—narrows in old age. Future studies need to revisit the age-as-leveller pattern and the cumulative advantage/disadvantage pattern by considering the multidimensional health outcomes in a more systematic way. In particular, future studies need to test, as suggested by this study, if the biologically-driven processes of health may correspond to the age-as-leveller pattern, whereas the culturally-driven dimensions of health may correspond to the cumulative advantage/disadvantage pattern in old age.

### Supplementary Information

**Additional file 1. Tabls S1:** Effects of income on alternative health outcomes as a function of age among older adults in Europe. **Table S2:** Effects of income on alternative health outcomes as a function of age among older adults in China. **Table S3:** Effects of income on multimorbidity as a function of age among older adults in Europe and China (the Additive Scale).

## Data Availability

Data may be obtained from a third party and are not publicly available. For more information on the SHARE dataset, please refer to http://www.share-project.org/data-access/user-registration.html; for more information on the CHARLS dataset, please refer to http://charls.pku.edu.cn/en/Data/Harmonized_CHARLS.htm. The R scripts to reproduce our findings are available via the Open Science Framework (OSF): https://osf.io/mb8nc/?view_only=b4d526e930594d66a7428db9fbefc4ba.

## Footnotes

1 In this occurrence, the term “effect” is used to refer to a statistical effect and does not denote a causal effect.

2 For SHARE, 71%-74% of participants remain in the same income quintile wave-to-wave; for CHARLS, 69%-74% of participants remain in the same income quintile wave-to-wave.

3 The 12 countries for which the age-as-leveller effect was observed were Austria, Belgium, Czech Republic, Denmark, Estonia, France, Germany, Greece, Hungary, Italy, Sweden, Switzerland; the 7 countries for which this effect was not statistically significant were Israel, Luxembourg, Netherlands, Poland, Portugal, Slovenia, Spain. We did not identify a clear pattern in the countries where the effect was observed or not. It is possible that the variations in the effect are due to insufficient power in the country-specific subsamples.
