# Supplementary material for: Evolution of the income-related gap in health with old age: evidence from 20 countries in European and Chinese panel datasets
Source: Eur J Ageing. 2023 Aug 10;20(1):33. doi: 10.1007/s10433-023-00781-y (PMC10415242; doi:10.1007/s10433-023-00781-y)
Supplement: Supplementary file 1 — Additional file 1. Tabls S1: Effects of income on alternative health outcomes as a function of age among older adults in Europe. Table S2: Effects of income on alternative health outcomes as a function of age among older adults in China. Table S3: Effects of income on multimorbidity as a function of age among older adults in Europe and China (the Additive Scale). [file 10433_2023_781_MOESM1_ESM.docx]

**Supplemental material for
Evolution of the income-related gap in health with old age:**

**evidence from 20 countries in European and Chinese panel datasets**

### **Table S1**

### *Effects of Income on Alternative Health Outcomes as a Function of Age among Older Adults in Europe*

|  | Functional Disability | | Mobility Disability | | Memory | |
| --- | --- | --- | --- | --- | --- | --- |
|  | IRRs | 95% CI | IRRs | 95% CI | IRRs | 95% CI |
| Grand-mean centered mean age | 2.49^***^ | 2.40–2.58 | 1.56^***^ | 1.55–1.58 | 0.88^***^ | 0.88–0.89 |
| Person-mean centered age | 7.44^***^ | 6.07–9.13 | 1.60^*^ | 1.01–2.53 | 0.97^***^ | 0.95–0.98 |
| Equivalized income decile (1 = *bottom 10%*, 10 = *top 10%*) | 0.59^***^ | 0.52–0.67 | 0.76^***^ | 0.73–0.79 | 1.08^***^ | 1.07–1.09 |
| Grand-mean centered mean age × equivalized income decile | 1.15^*^ | 1.03–1.28 | 1.14^***^ | 1.10–1.18 | 1.04^***^ | 1.03–1.05 |
| Middle-aged adults (-2 SD) | 0.46^***^ | 0.35–0.61 | 0.60^***^ | 0.56–0.64 | 1.00 | 0.99–1.02 |
| Older middle-aged adults (-1 SD) | 0.52^***^ | 0.43–0.63 | 0.68^***^ | 0.64–0.71 | 1.04^***^ | 1.03–1.05 |
| Older adults (+1 SD) | 0.67^***^ | 0.59–0.76 | 0.86^***^ | 0.82–0.90 | 1.12^***^ | 1.11–1.13 |
| Oldest old adults (+2 SD) | 0.76^**^ | 0.63–0.91 | 0.96 | 0.90–1.04 | 1.16^***^ | 1.14–1.18 |
| Person-mean centered age × equivalized income decile | 1.23 | 0.97–1.55 | 1.10^***^ | 1.05–1.15 | 1.04^***^ | 1.02–1.06 |
| Earlier panel waves (-2 SD) | / | / | 0.72^***^ | 0.69–0.76 | 1.05^***^ | 1.04–1.07 |
| Early panel waves (-1 SD) | / | / | 0.74^***^ | 0.71–0.77 | 1.07^***^ | 1.06–1.08 |
| Late panel waves (+1 SD) | / | / | 0.78^***^ | 0.76–0.81 | 1.09^***^ | 1.08–1.10 |
| Later panel waves (+2 SD) | / | / | 0.81^***^ | 0.77–0.84 | 1.10^***^ | 1.09–1.12 |
| Wealth decile (1 = *bottom 10%*, 10 = *top 10%*) | 0.49^***^ | 0.44–0.55 | 0.59^***^ | 0.57–0.61 | 1.07^***^ | 1.07–1.08 |
| Upper secondary or vocational education | 0.63^***^ | 0.59–0.68 | 0.83^***^ | 0.81–0.85 | 1.13^***^ | 1.12–1.13 |
| Tertiary education | 0.50^***^ | 0.46–0.55 | 0.66^***^ | 0.64–0.68 | 1.21^***^ | 1.20–1.22 |
| Gender (-0.5 = *men*, +0.5 = *women*) | 0.89^***^ | 0.84–0.94 | 1.51^***^ | 1.48–1.54 | 1.07^***^ | 1.06–1.07 |
| Region of residence (0 = *urban*, 1 = *rural*) | 1.02 | 0.96–1.07 | 1.01 | 1.00–1.03 | 1.00 | 0.99–1.00 |
| Current marital status (0 = *not married*, 1 = *married*) | 0.79^***^ | 0.74–0.84 | 0.94^***^ | 0.92–0.96 | 1.03^***^ | 1.03–1.04 |
| Current working status (0 = *not working*, 1 = *working*) | 0.50^***^ | 0.45–0.55 | 0.76^***^ | 0.75–0.78 | 1.02^***^ | 1.01–1.02 |
| Household size | 1.18^***^ | 1.15–1.21 | 1.02^***^ | 1.01–1.03 | 0.99^***^ | 0.99–0.99 |
| *N*_participants_ | 73,407 |  | 73,407 |  | 73,166 |  |
| Observations | 242,753 |  | 242,948 | | 237,317 | |

*Note.* IRRs = incidence rate ratios. Comparisons were made between the bottom 10% and the top 10% in terms of income and wealth.

^*^*p <* .05. ^**^*p <* .01. ^***^*p <* .001

### **Table S2**

### *Effects of Income on Alternative Health Outcomes as a Function of Age among Older Adults in China*

|  | Functional Disability | | Mobility Disability | | Memory | |
| --- | --- | --- | --- | --- | --- | --- |
|  | IRRs | 95% CI | IRRs | 95% CI | IRRs | 95% CI |
| Grand-mean centered mean age | 1.63^***^ | 1.57–1.70 | 1.32^***^ | 1.29–1.35 | 0.87^***^ | 0.86–0.88 |
| Person-mean centered age | 2.39^***^ | 2.03–2.80 | 1.87^***^ | 1.80–1.94 | 0.58^***^ | 0.57–0.60 |
| Equivalized income decile (1 = *bottom 10%*, 10 = *top 10%*) | 0.46^***^ | 0.40–0.53 | 0.64^***^ | 0.60–0.69 | 1.21^***^ | 1.17–1.24 |
| Grand-mean centered mean age × equivalized income decile | 1.32^***^ | 1.17–1.49 | 1.08^*^ | 1.01–1.16 | 1.11^***^ | 1.08–1.14 |
| Middle-aged adults (-2 SD) | 0.27^***^ | 0.20–0.35 | 0.55^***^ | 0.48–0.64 | 1.00 | 0.95–1.05 |
| Older middle-aged adults (-1 SD) | 0.34^***^ | 0.28–0.41 | 0.60^***^ | 0.54–0.66 | 1.10^***^ | 1.06–1.13 |
| Older adults (+1 SD) | 0.57^***^ | 0.49–0.67 | 0.69^***^ | 0.63–0.76 | 1.32^***^ | 1.27–1.38 |
| Oldest old adults (+2 SD) | 0.74^*^ | 0.58–0.94 | 0.74^***^ | 0.64–0.85 | 1.46^***^ | 1.37–1.55 |
| Person-mean centered age × equivalized income decile | 1.41 | 0.80–2.47 | 1.17^*^ | 1.03–1.32 | 1.38^***^ | 1.27–1.51 |
| Earlier panel waves (-2 SD) | / | / | 0.59^***^ | 0.54–0.66 | 1.02 | 0.98–1.07 |
| Early panel waves (-1 SD) | / | / | 0.62^***^ | 0.57–0.67 | 1.11^***^ | 1.08–1.14 |
| Late panel waves (+1 SD) | / | / | 0.67^***^ | 0.62–0.72 | 1.30^***^ | 1.25–1.35 |
| Later panel waves (+2 SD) | / | / | 0.69^***^ | 0.63–0.76 | 1.41^***^ | 1.34–1.49 |
| Wealth decile (1 = *bottom 10%*, 10 = *top 10%*) | 0.55^***^ | 0.49–0.62 | 0.65^***^ | 0.60–0.69 | 1.08^***^ | 1.05–1.10 |
| Upper secondary or vocational education | 0.47^***^ | 0.39–0.56 | 0.76^***^  0.70 – 0.82 | 0.70–0.82 | 1.22^***^ | 1.19–1.25 |
| Tertiary education | 0.34^***^ | 0.19–0.62 | 0.64^***^ | 0.52–0.80 | 1.29^***^ | 1.22–1.37 |
| Gender (-0.5 = *men*, +0.5 = *women*) | 1.81^***^ | 1.69–1.93 | 1.66^***^ | 1.60–1.73 | 0.95^***^ | 0.93–0.96 |
| Region of residence (0 = *urban*, 1 = *rural*) | 1.37^***^ | 1.27–1.48 | 1.14^***^ | 1.09–1.18 | 0.92^***^ | 0.90–0.93 |
| Current marital status (0 = *not married*, 1 = *married*) | 1.09 | 1.00–1.18 | 1.01 | 0.96–1.05 | 1.07^***^ | 1.05–1.09 |
| Current working status (0 = *not working*, 1 = *working*) | 0.80^***^ | 0.75–0.85 | 0.80^***^ | 0.78–0.82 | 1.00 | 0.99–1.02 |
| Household size | 1.03^***^ | 1.01–1.05 | 1.01^**^ | 1.00–1.02 | 0.99^*^ | 0.99–0.99 |
| *N*_participants_ | 9,999 |  | 10,063 |  | 10,006 |  |
| Observations | 26,106 |  | 36,307  , | | 33,045 | |

*Note.* IRRs = incidence rate ratios. Comparisons were made between the bottom 10% and the top 10% in terms of income and wealth.

^*^*p <* .05. ^**^*p <* .01. ^***^*p <* .001

### **Table S3**

### *Effects of Income on Multimorbidity as a Function of Age among Older Adults in Europe and China (the Additive Scale)*

|  | Europe | | China | |
| --- | --- | --- | --- | --- |
| Between-participant effect |  |  |  |  |
| RERI | 0.11 [0.09, 0.12] | | 0.18 [0.15, 0.21] | |
| AP | 0.08 [0.07, 0.09] | | 0.15 [0.13, 0.17] | |
| SI | 1.69 [1.59, 1.78] | | 12.25 [2.16, 22.49] | |
| Within-participant effect |  |  |  |  |
| RERI | 0.17 [0.10, 0.24] | | 0.12 [-0.14, 0.38] | |
| AP | 0.08 [0.05, 0.11] | | 0.05 [-0.05, 0.15] | |
| SI | 1.18 [1.11, 1.26] | | 1.09 [0.91, 1.30] | |
| *Note*. RERI = relative excess risk due to interaction. AP = attributable proportion.  SI = synergy index. | | | | |
